# Intention to utilize mental health and suicide prevention resources in a community sample during the first year of the COVID-19 pandemic

**DOI:** 10.1186/s12888-025-06492-1

**Published:** 2025-01-29

**Authors:** Lisa J. Cohen, Rawad El Hayek, Benedetta Imbastaro, Inna Goncearenco, Sifan Zheng, Megan L. Rogers, Maurizio Pompili, Igor Galynker

**Affiliations:** 1 Department of Psychiatry, Icahn School of Medicine at Mount Sinai, Mount Sinai Behavioral Health Center, New York, NY USA; 2 Psychiatry Residency Program, New York Medical College at Westchester Medical Center, Valhalla, NY USA; 3 Department of Psychology, Texas State University, San Marcos, TX USA; 4 Dept. of Neurosciences, Mental Health and Sensory Organs. Sant’Andrea Hospital, Sapienza University of Rome, Rome, Italy

**Keywords:** Suicide prevention, Suicide crisis syndrome, Covid-19 pandemic.

## Abstract

**Objective:**

Given the stressors experienced during the COVID-19 pandemic, it is critical to identify populations with elevated mental health needs during this crisis. This study investigated demographic correlates of reported intention to utilize mental health (MH) and suicide prevention (SP) resources in a community sample during the COVID-19 pandemic.

**Methods:**

A sample of 1,978 adults in the United States completed an anonymous online survey between June 2020 and February 2021.

**Results:**

Intent to utilize MH resources was associated with younger age, single marital status, female gender, and Hispanic vs. White race/ethnicity. Intent to utilize SP resources was associated with younger age, single marital status, and was greater among Black and Hispanic vs. White race/ethnicity. Lower education was associated with MH and SP utilizers in bivariate analysis. Indirect effects of Suicide Crisis Syndrome (SCS) symptoms were found on the association of age, gender, and marital status with MH utilization and of age, marital status, and education with SP Utilization.

**Conclusions:**

Specific demographic populations demonstrate greater interest in mental health care during the COVID-19 pandemic. These help-seeking patterns can be explained in part by an elevated level of SCS symptoms, suggesting greater levels of distress were driving expressed intention to utilize service referrals.

The widespread mental health sequelae of the COVID-19 pandemic are well documented [1, 2]. The COVID-19 pandemic exposed individuals to multiple stressors in the United States as well as globally, adversely affecting all aspects of life. Factors like social isolation, financial insecurity, fear of being infected, and loss of loved ones contributed to the increased prevalence of anxiety, depression, PTSD, psychological distress and suicidal thinking [2].

Importantly, however, the effects of the pandemic were not uniform, affecting some groups more than others. Within the United States, certain ethnic minority groups, such as African, Hispanic, and Asian Americans had higher rates of COVID-19-related hospitalization, deaths, depression, anxiety, and suicidal ideation (SI) compared to White/European Americans [3, 4]. In this context, as well as in light of longstanding demographic disparities in access to health care in the United States [5–7], it is important to identify segments of the population who express greater interest in mental health care. It is also important to investigate the extent to which such interest can be explained by increased emotional distress, rather than to traditional help-seeking patterns and inequities alone.

## Help-seeking behavior barriers

Despite the high prevalence of treatable and preventable mental health disorders, nearly two-thirds of individuals struggling with them do not receive treatment [8–12]. Likewise, only about a third of individuals who died by suicide sought professional mental health resources within the 12 months before their deaths [13–15]. Clearly, the relationship between psychiatric need and help-seeking behavior is complex.

Research has identified sociodemographic differences in mental health help-seeking behavior, with female gender [16], younger age [17] and a lack of partner [18] being the most consistent correlates of help-seeking. Findings on level of education and help-seeking behaviors have been inconsistent, with some studies suggesting an inverse relationship [19, 20] while others a direct one [7]. Importantly, ethnic minorities have been shown to face more structural and attitudinal barriers compared with the White population. A recent review demonstrated that racial minorities in the U.S. were more likely to experience overt and covert racial discrimination and inadequate healthcare, compared to the White population [6]. Structural barriers include clinicians’ implicit racial biases that negatively impact patient-provider interaction, referral and treatment decisions [5, 21]. Furthermore, racial discrimination experienced by minority communities contributes to an ongoing lack of trust in the mental health care system, thus creating attitudinal barriers which further hinder help-seeking behaviors [22].

## Help-seeking barriers regarding suicide risk

Of all mental health difficulties, the most serious involves suicide risk. The most recent CDC report (2022) indicated that the overall annual U.S. suicide rate increased 30% between 2000 and 2020. Therefore, it is of particular importance to identify those most in need of mental health care for suicide prevention while keeping in mind barriers to help-seeking behavior. A lack of perceived need for services, preference for self-management, fear of hospitalization, and structural factors (i.e., time constraints, finances) have been posited as key barriers to mental health care seeking among suicidal individuals [23]. A more recent review identified minority ethnicity, lower psychological distress, no mental health diagnosis, and lower severity of suicidality as predictive of not seeking care among people at risk for suicide [24].

## Suicide crisis syndrome

Given the differing levels of stress experienced during the pandemic across different demographic groups, it is also of interest to examine whether acute and stress-induced pre-suicidal states might account for the relationship between sociodemographic factors and help-seeking behavior. If so, this would indicate that demographic differences in help seeking behavior may be due to increased emotional distress during the pandemic rather than solely to longstanding barriers/facilitators to health care. Such a pre-suicidal state exists: recent research has introduced the notion of an acute state that is characterized by intense negative affect and hyperarousal and that can be triggered by stressful life events, such as the COVID-19 pandemic [25–28].

Specifically, the Suicide Crisis Syndrome (SCS; [25]), an acute hyperaroused, negative affect state, driven by a sense of entrapment, is predictive of imminent suicidal thoughts and behavior [29, 30]. The SCS is characterized by two criteria: Frantic Hopelessness/Entrapment (Criterion A) and Associated Disturbances (Criteria B), which is composed of Affective Disturbance (B1), Loss of Cognitive Control (B2), Hyperarousal (B3), and Acute Social Withdrawal (B4). Accumulated research has shown its concurrent validity in relation to current and lifetime suicidality [29, 31, 32] and its predictive validity in relation to short-term suicidal behavior [33, 34] above and beyond SI and other relevant risk factors [35]. Likewise, in a previous analysis of the same dataset used here, [36] (Under Review) showed that an SCS diagnosis correlated with greater help-seeking behavior regarding both mental health and suicide prevention resources during the COVID-19 pandemic.

In the present study, we evaluated the predictors of help-seeking intentions within a large community-based convenience sample studied in an online survey during the first year of the COVID-19 pandemic. Specifically, we investigated demographic correlates of reported intention to utilize mental health and suicide prevention resources provided in the survey. Additionally, we explored the potentially mediating role of the SCS in determining patterns of help-seeking intentions.

We specifically addressed three research questions:

1. Which demographic groups were most likely to express intention to pursue mental health or suicide prevention service referrals?
2. Are these patterns consistent with previously documented patterns of health care access and help seeking behavior?
3. Can patterns of help seeking intentions be explained by differential levels of emotional distress, specifically higher symptoms of the SCS?

We hypothesized that the presence of the SCS would account for the impact of demographic predictors on intended resource utilization. To the extent that greater SCS symptoms can explain heightened help-seeking intentions in certain subgroups, we can attribute these patterns to increased psychiatric need rather than various facilitators/barriers to care.

## Methods

### Subjects

Study participants included 1,978 survey respondents, recruited between June 2020 and February 2021 through online paid advertisements and postings on social media. The only exclusion criterion was age less than 18. This survey comprised the United States component of a large international survey, administered across 11 different countries, and specifically focused on suicide risk factors during the COVID pandemic. The present analyses were restricted to the United States sample to facilitate interpretation of socio-demographic information and avoid the potentially confounding effect of international differences in pandemic severity and related governmental responses. Further, data on the U.S. sample were collected in the first year of the pandemic, prior to the wide availability of vaccines.

The survey was described to participants as evaluating the mental health effects of the COVID-19 pandemic, specifically symptoms of distress, anxiety, or SI. Interested participants were directed to a secure online platform (Qualtrics) where they provided electronic consent and completed an anonymous battery of sociodemographic and clinical questionnaire. Participants were entered into a raffle for one of thirty $15 gift cards. All study procedures were approved by the Icahn School of Medicine at Mount Sinai Institutional Review Board.

### Measures

#### Resource utilization questions

Towards the end of the survey, a list of mental health and suicide prevention resources were presented via a linked document containing a series of relevant hotlines and websites, a link to locate nearby hospitals, and contact information for the principal investigator.

Follow-up questions addressed participants’ intention to utilize these resources and their reasons for doing so. This was done with the following questions: “Do you plan to utilize any of the mental health resources?”, “Do you plan to utilize any of the suicide prevention resources?” The first 502 subjects in the study were instructed to answer yes, no, or maybe to these questions. The next 1,476 subjects were provided with five response options, four “Yes” responses associated with a specific reason (for personal use, for someone I know, for my own education, and for another reason) and a single “no” response. In the current study, all patients who provided at least one yes response in either format were coded as “utilizers,” i.e., as endorsing intention to utilize either mental health (MH) or suicide prevention (SP) resources, respectively. All other participants were rated as “non-utilizers” for either MH or SP resources.

#### Suicide crisis inventory—2 (SCI-2; [33])

The SCI-2 is a 61-item self-report scale that was revised from the original SCI (Galynker et al., 2017) to better reflect the five criteria for the SCS: entrapment (10 items), affective disturbances (18 items), loss of cognitive control (15 items), hyperarousal (13 items), and social withdrawal (5 items). Respondents indicated the degree to which they experienced each symptom based on their worst feelings over the last several days on a 5-point scale ranging from 0 (Not at all) to 4 (Extremely), with select items reverse coded such that higher scores reflect more severe SCS symptoms. The SCI-2 has demonstrated evidence of internal consistency, convergent and discriminant validity, and predictive validity in relation to suicide attempts in past research [33]. Internal consistency in this sample was very high (α = 98).

### Data analytic strategy

To investigate the association between demographic characteristics and the stated intention to utilize either mental health or suicide prevention resources, bivariate logistic regressions were conducted, using IBM SPSS statistics version 27. Predictors included five demographic variables (age, gender, race/ethnicity, marital status, education, and work status). Associations between five potential clinical confounds (currently in psychotherapy, group therapy or on psychiatric medication; prior psychiatric hospitalization) and MH and SP status were also assessed by bivariate logistic regressions. Two multiple logistic regressions were performed next, one for MH and SP resources, respectively, with all significant predictors in bivariate analyses entered either as independent variables or covariates. To assess the role of the SCS in accounting for the statistically significant results found in the above analyses, indirect effects analyses were conducted using PROCESS version 4.0 [37], according to methods suggested by Hayes [38].

## Results

### Demographic and clinical characteristics

The sample was composed of 1,978 participants who completed all relevant instruments, 87% of whom identified as female, with a mean age of 27.56 ± 9.6 years. The most common ethnicity was White (83%) followed by Hispanic (8%), Asian (4%) and Black (3%). The most frequent marital status was never married/single (54%), followed by steady relationship (23%) and married (12%). The majority of the sample completed a 4-year college education (67.0%) and 17.5% had graduate level education. With regard to work status, 54% reported having a full-time job, 13% had a part-time job, 21% described themselves as full-time students, and 9% as unemployed.

Concerning current clinical mental health treatment, 24% of the sample reported being in individual therapy, 1% in group therapy, and 27% in psychopharmacological treatment. Seven percent (7%) of participants reported having been psychiatrically hospitalized at least once and 33% admitted denying or concealing SI to a doctor or clinician in the past. Notably, a large majority (1,275, 64.5%) reported lifetime history of SI and a large minority reported SI in the past month (577, 29.3%). A smaller subset endorsed more serious SI, including method, intent and or plan: 598 (30.3%) for lifetime and 172 (8.7%) in the past month.

### Mental health and suicide prevention resources

Over one third (36%) of the sample endorsed intention to use mental health resources (MH utilizers) and 20% to use suicide prevention resources (SP Utilizers). Among the participants who were asked for their reason for utilizing the resources, almost 2/3 of the MH utilizers (63%) stated an interest in MH resources for their own “personal use,” though only about a third (38%) stated the same reason for SP resources. Among the SP utilizers, the most frequent reason given was “for my own education” (49%). The vast majority of either MH or SP utilizers reported seeking out these resources for themselves; only 4.5% and 11% (for MH and SP, respectively) reported intending these resources *only* for someone else and not themselves.

### Associations between demographic characteristics and the stated intention to utilize mental health (MH) resources

To investigate the unadjusted associations between sociodemographic characteristics and the stated intention to utilize MH resources, we conducted bivariate logistic regressions (See Table 1). Age was inversely related with intention to utilize MH resources, such that younger participants were more likely to do so than older ones. Self-identified women were almost twice as likely to utilize MH resources compared to self-identified men. The cell sizes were not sufficient to detect differences among other gender categories. Regarding race/ethnicity, Hispanic participants reported higher help-seeking intentions in comparison with White participants, whereas Asian subjects reported significantly less intentions to seek MH resources. In comparison with never married subjects, married participants reported less inclination to use MH resources. Likewise, a higher education level correlated with lower help-seeking intentions; those achieving a graduate level education were less inclined to use MH resources than those with a high-school education. No significant differences were found between MH utilizers and non-utilizers with regard to work status.

**Table 1:** Demographic and Clinical Correlates of Reported Intention to Utilize Mental Health Resources: Bivariate and Multiple Logistic Regressions

Predictor	No N (%) 1262 (63.8)	Yes N (%) 716 (36.2)	Odds Ratio	*P* value	95% CI Lower Limit	95% CI Upper limit	Odds Ratio	P value	95% CI Lower Limit	95% CI Upper limit
			**Bivariate Analyses**				**Multivariate Analysis**			
**Age**										
Age (Mean ± s.d.)	27.56 ± 9.6	24.88 ± 5.3	**0.95**	< 0.001***	0.94	0.97	**0.96**	< .001***	0.94	0.98
**Gender**										
Male	167 (75.9)	53 (24.1)	Ref	–-	–-	–-	Ref	–-	–-	–-
Female	1067 (61.9)	657 (38.1)	**1.94**	< 0.001***	1.40	2.68	**1.65**	.004**	1.17	2.33
Trans M to F	4 (66.7)	2 (33.3)	1.58	.606	0.28	8.85	1.19	.843	0.21	6.86
Trans F to M	6 (100)	0 (0.0)	0.00	.999	0.00		0.00	.999	0.00	
Trans not binary	14 (82.4)	3 (17.6)	0.68	.549	0.19	2.44	0.65	.521	0.18	2.40
Not Sure	2 (100)	0 (0.0)	0.00	.999	0.00		0.00	.999	0.00	
Decline	2 (66.7)	1 (33.3)	1.58	.713	0.14	17.72	2.10	.572	0.16	27.65
**Race/Ethnicity**										
White	1957 (64.3)	587 (35.7)	Ref	–-	–-	–-	Ref	–-	–-	–-
Other/declined	16 (88.9)	2 (11.1)	**0.23**	.047*	0.05	0.98	0.26	.077	0.06	1.16
Black	34 (54.8)	28 (45.2)	1.48	.130	0.89	2.47	1.64	.074	0.95	2.81
Native Amer	12 (63.2)	7 (36.8)	1.05	.918	0.41	2.68	1.09	.856	0.42	2.86
Hispanic	77 (51.0)	74 (49.0)	**1.73**	.001**	1.24	2.42	**1.63**	.005**	1.16	2.29
Asian^1^	66 (78.6)	18 (21.4)	**0.49**	.009**	0.29	0.84	**0.52**	.017*	0.30	0.89
**Marital Status**										
Never Married	656 (61.7)	407 (38.3)	Ref	–-	–-	–-	Ref	–-	–-	–-
Married	183 (80.3)	45 (19.7)	**0.40**	< 0.001***	0.28	0.56	**0.64**	.022*	0.43	0.94
Separated	12 (70.6)	5 (29.4)	0.67	.458	0.24	1.92	1.11	.848	0.37	3.35
Divorced	25 (62.5)	15 (37.5)	0.97	.920	0.50	1.86	2.12	.051	0.996	4.49
Widowed	4 (80.0)	1 (20.0)	0.40	.417	0.05	3.62	1.59	.706	0.14	17.80
Steady relationship	280 (62.2)	170 (37.8)	0.98	.852	0.78	1.23	0.95	.694	0.76	1.21
Cohabitating	102 (58.3)	73 (41.7)	1.15	.389	0.83	1.60	1.21	.274	0.86	1.69
**Education**										
High School	47 (56.0)	37 (44.0)	Ref	–-	–-	–-	Ref	–-	–-	–-
2-year College	89 (54.6)	74 (45.4)	1.06	.840	0.62	1.79	1.09	.756	0.63	1.89
Some College	241 (59.4)	165 (40.6)	0.87	.564	0.54	1.40	0.86	.538	0.52	1.40
4-year College	641 (65.5)	337 (34.5)	0.67	.079	0.43	1.05	0.76	.241	0.47	1.21
Masters	199 (69.3)	88 (30.7)	**0.56**	.023*	0.34	0.93	0.79	.382	0.47	1.34
Doctorate	45 (75.0)	15 (25.0)	**0.42**	.020*	0.21	0.88	0.71	.380	0.33	1.53
**Work Status**^**2**^										
Unemployed	126 (67.7)	60 (32.3)	Ref				–-	–-	–-	–-
Part-time	167 (63.3)	97 (36.7)	1.22	.326	0.82	1.81	–-	–-	–-	–-
Disabled	9 (75.0)	3 (25.0)	0.70	.603	0.18	2.68	–-	–-	–-	–-
Volunteer	7 (77.8)	2 (22.2)	0.60	.532	0.12	2.98	–-	–-	–-	–-
Retired	15 (88.2)	2 (11.8)	0.28	.098	0.06	1.26	–-	–-	–-	–-
Full-Time Housework	17 (77.3)	5 (22.7)	0.62	.365	0.22	1.75	–-	–-	–-	–-
Full-Time Job	656 (61.9)	404 (38.1)	1.29	.128	0.93	1.80	–-	–-	–-	–-
Full-Time Stud	265 (65.0)	143 (35.0)	1.13	.506	0.78	1.64	–-	–-	–-	–-

*Note*. *p < .05, **p < .01, ***p < .001, Bonferroni corrected alpha for bivariate analyses = .05/6 = .008, ^1^ including Pacific Islands,^2^ ongoing at time of survey

All variables significant in bivariate analyses were then entered into a multivariable logistic regression analysis. The results were largely consistent with the previous analyses; younger age, female gender, and Hispanic race/ethnicity were predictive of MH utilizer status while Asian race/ethnicity and being married remained predictive of non-utilizer status. The effect of education did not retain significance (see Table 2).

**Table 2:** Demographic and Clinical Correlates of Reported Intention to Utilize Suicide Prevention Resources – Bivariate and Multiple Logistic Regressions

Predictor	No N (%) 1583 (80.0)	Yes N (%) 395 (20.0)	Odds Ratio Exp(B)	P value	95% CI Lower Limit	95% CI Upper limit		Odds Ratio Exp(B)	*P* value	95% CI Lower Limit		95% CI Upper limit
			**Bivariate Analyses**					**Multivariate Analyses**				
**Age**												
Age (Mean ± s.d.)	27.07 ± 8.9	24.65 ± 5.6	**0.95**	< 0.001***	0.93		0.97	**0.96**	.001**	0.94	0.98	
**Gender**												
Male	186 (84.5)	34 (15.5)	Ref	–-	–-		–-	–-	–-	–-	–-	
Female	1367 (79.3)	357 (20.7)	1.43	.068	0.97		2.10	–-	–-	–-	–-	
Trans male to female	5 (83.3)	1 (16.7)	1.09	.935	0.12		9.66	–-	–-	–-	–-	
Trans female to male	6 (100)	0 (0.0)	0.00	.999	0.00			–-	–-	–-	–-	
Trans non binary	14 (82.4)	3 (17.6)	1.17	.811	0.32		4.30	–-	–-	–-	–-	
Not Sure	2 (100)	0 (0.0)	0.00	.999	0.00			–-	–-	–-	–-	
Declined to answer	3 (100)	0 (0.0)	0.00	.999	0.00			–-	–-	–-	–-	
**Race/Ethnicity**												
White	1336 (81.3)	308 (18.7)	Ref	–-	–-		–-	Ref	–-	–-	–-	
Other/declined	15 (83.3)	3 (16.7)	0.87	.823	0.25		3.02	1.03	.961	0.29	3.70	
Black	43 (69.4)	19 (30.6)	**1.92**	.021*	1.10		3.34	1.72	.069	0.96	3.10	
Native American	15 (78.9)	4 (21.1)	1.16	.797	0.38		3.51	1.11	.862	0.36	3.42	
Hispanic	105 (69.5)	46 (30.5)	**1.90**	.001**	1.32		2.75	**1.80**	.002**	1.24	2.63	
Asian^1^	69 (82.1)	15 (17.9)	0.94	.841	0.53		1.67	0.93	.805	0.52	1.68	
**Marital Status**												
Nev Married	828 (77.9)	235 (22.1)	Ref	–-	–-		–-	Ref	–-	–-	–-	
Married	205 (89.9)	23 (10.1)	**0.40**	< 0.001***	0.25		0.62	0.65	.088	0.39	1.07	
Separated	16 (94.1)	1 (5.9)	0.22	.143	0.03		1.67	0.31	.266	0.04	2.43	
Divorced	32 (80.0)	8 (20.0)	0.88	.752	0.40		1.94	1.59	.298	0.66	3.83	
Widowed	4 (80.0)	1 (20.0)	0.88	.910	0.10		7.92	4.88	.206	0.42	56.79	
Steady relationship	363 (80.7)	87 (19.3)	0.84	.229	0.64		1.11	0.86	.306	0.65	1.15	
Cohabitating	135 (77.1)	40 (22.9)	1.04	.825	0.71		1.53	1.10	.633	0.74	1.64	
**Education**												
High School	61 (72.6)	23 (27.4)	Ref	–-	–-		–-	Ref	–-	–-	–-	
2-year College	108 (66.3)	55 (33.7)	1.35	.309	0.76		2.41	1.52	.174	0.83	2.80	
Some College	306 (75.4)	100 (24.6)	0.87	.597	0.51		1.47	0.92	.768	0.53	1.61	
4-year College	817 (83.5)	161 (16.5)	**0.52**	.012*	0.31		0.87	0.68	.161	0.40	1.17	
Master	238 (82.9)	49 (17.1)	**0.55**	.037*	0.31		0.97	0.86	.628	0.47	1.58	
Doctorate	53 (88.3)	7 (11.7)	**0.35**	.026*	0.14		0.88	0.64	.364	0.24	1.68	
**Work Status**^**2**^												
Unemployed	154 (82.8)	32 (17.2)	Ref	–-	–-		–-	–-	–-	–-	–-	
Part-time	211 (79.9)	53 (20.1)	1.21	.444	0.74		1.96	–-	–-	–-	–-	
Disabled	10 (83.3)	2 (16.7)	0.96	.962	0.20		6.55	–-	–-	–-	–-	
Volunteer	8 (88.9)	1 (11.1)	0.60	.637	0.07		4.98	–-	–-	–-	–-	
Retired	15 (88.2)	2 (11.8)	0.64	.568	0.14		2.95	–-	–-	–-	–-	
Full-Time Housework	18 (81.8)	4 (18.2)	1.07	.909	0.34		3.37	–-	–-	–-	–-	
Full-Time Job	320 (78.4)	88 (21.6)	1.21	.361	0.80		1.82	–-	–-	–-	–-	
Full-Time Student	1292 ( 93.7)	87 (6.3)	1.32	.220	0.85		2.07	–-	–-	–-	–-	

*Note*. All analyses are bivariate logistic regressions, *p < .05, **p < .01, ***p < .001, Bonferroni corrected alpha for bivariate analyses = .05/6 = .008, ^1^ including Pacific Islands, ^2^ongoing at time of survey

### Associations between demographic characteristics and intention to utilize suicide prevention (SP) resources

The same analyses were conducted to assess the relationships between the demographic characteristics and stated intention to utilize SP resources (see Table 3). Similar to the MH findings, in bivariate analyses, older age and higher level of education were inversely related to intention to utilize SP resources. Being married was also associated with decreased intention to utilize these resources. Interestingly, the association with race/ethnicity was somewhat different. Participants who identified as Black or Hispanic reported greater intention to utilize SP resources. No significant associations were found regarding gender or work status.

**Table 3:** Indirect Effect of SCS on the Relationship between Demographic and Clinical Variables and Reported Intention to Utilize Mental Health and Suicide Prevention Resources

**Independent** **variable**	**Indirect effect**				**Direct effect**					
	**Effect**	**S.E.**^2^	**LLCI**^2^	**ULCI**^2^	**Effect**	**S.E.**^2^	**Z/t score**	***P* value**	**LLCI**^2^	**ULCI**^2^
**Mental Health Resources**										
**Age**	**-0.01**	0.001	-0.01	-0.004	-0.04	0.01	-5.55	**<0.001**	-0.06	-0.03
**Gender ** Female vs. Male	**0.14**	0.03	0.09	0.21	0.54	0.17	3.20	**.001**	0.21	0.87
**Race ** Hispanic vs. White	-0.001	0.01	-0.02	0.02	-0.19	0.06	-3.23	**.001**	-0.30	-0.07
**Race ** Asian^1^ vs. White	0.01	0.02	-0.04	0.05	0.36	0.14	2.63	**.009**	0.09	0.63
**Marital Status** Married vs. Never Married	**-0.09**	0.03	-0.16	-0.04	-0.85	0.18	-4.73	**<0.001**	-1.20	-0.50
**Education** Masters vs. High School	-0.01	0.02	-0.05	0.03	-0.14	0.07	-2.05	**.040**	-0.27	-0.006
**Education** Doctorate vs. High School	-0.003	0.03	-0.06	0.04	-0.17	0.08	-2.18	**.030**	-0.32	-0.02
**Suicide Prevention Resources**										
**Age**	**-0.01**	0.002	-0.01	-0.007	-0.04	0.01	-3.90	**<0.001**	-0.06	-0.02
**Race** Black vs. White	-0.02	0.02	-0.05	0.01	-0.11	0.05	-1.90	.057	-0.23	0.003
**Race** Hispanic vs. White	-0.002	0.01	-0.03	0.03	-0.22	0.06	-3.44	**<0.001**	-0.35	-0.01
**Marital Status** Married vs. Never Married	**-0.14**	0.04	-0.22	-0.06	-0.82	0.24	-3.48	**<0.001**	-1.28	-0.36
**Education** 4-y College vs. High School	**-0.08**	0.03	-0.13	-0.03	-0.14	0.09	-1.56	.118	-0.32	-0.04
**Education** Masters vs. High School	-0.04	0.03	-0.10	0.001	-0.11	0.08	-1.40	.162	-0.26	0.04
**Education** Doctorate vs. High School	-0.04	0.04	-0.12	0.02	-0.17	0.10	-1.75	.081	-0.36	0.02

*Note*. *S.E.* Standard error, *LLCI* Lower limit confidence interval, *ULCI* Upper limit confidence interval

To assess the unique contribution of each significant predictor from bivariate analyses, multiple logistic regression was performed (See Table 2). A significant association was found between reported intention to utilize SP resources and younger age and Hispanic race/ethnicity. The significant effects of marital status, Black race/ethnicity and education were not retained after controlling for other relevant demographic factors.

### The role of SCS as an explanatory factor

In order to evaluate if patterns of help-seeking can be explained by the severity of SCS symptoms, we conducted a series of indirect effects analyses [38]. The significant predictor variables in bivariate logistic regression analyses were entered separately as the independent variable for each indirect effects analysis. The outcome variable was utilization group for either MH or SP resources and the explanatory variable was the SCI total score.

In the prediction to MH utilization group, we found significant indirect effects for age, gender, and marital status. This suggests that elevated SCS symptoms accounted, at least in part, for the relationship between these demographic groups and MH resource utilization (see Table 3). In the prediction to SP utilization group, age, marital status, and education (4-year college vs. high school) had significant indirect effects through SCS symptoms. Of note, the direct effects were significant for all independent variables in the MH model and for age and marital status but not education in the SP model.

## Discussion

The purpose of this study was to identify predictors of help-seeking behavior during the COVID-19 pandemic, specifically in relation to the intention to utilize mental health and suicide prevention services. Our findings suggest several sociodemographic factors that may influence help-seeking responses in this context. Additionally, our results showed significant indirect effects of the SCS on the relationship between specific sociodemographic factors and help-seeking intentions, highlighting the role of increased distress rather than health care access disparities alone in accounting for these patterns.

It is of note that the sample reported a remarkably high rate of both lifetime and recent suicidal ideation, with 64% reporting all kinds of lifetime SI and 30% reporting more serious SI, inclusive of either method, plan or intent. Almost a third of the sample (29.3%) reported SI in the past month, with 8.7% endorsing more serious SI. This is higher than is found in the general population although elevated rates are also found among specific subsamples, such as sexual minorities (LGBT +) [39] and Native Americans [40, 41]. In 2021, according to the CDC, 12.3 million adults endorsed serious suicidal thoughts and 3.5 million made suicide plans [42], which translates to about 4% and 1.1% of the U.S. population, respectively. Likewise, in an Australian epidemiological study, 16.5% of the sample reported lifetime suicidal ideation and 7.5% made plans [43]. Past year rates were 3.3% and 1.1%, respectively.

Our surprisingly high rate of SI is likely due to multiple causes, including a possible self-selection bias of people choosing to participate in a study described as “evaluating the mental health effects of the COVID-19 pandemic, specifically symptoms of distress, anxiety, or SI.” The methods of assessing SI may also differ across studies, inflating our own findings. Nevertheless, the study was conducted during the height of the pandemic and may have reflected its pathogenic impact. Similarly, the Arya study found an almost 50% increase in past-year SI rates between 2007 and 2020–2022, growing from 2.3% to 3.3%.

### Intention to utilize mental health services

Our study found that younger participants were more inclined to utilize mental health referrals compared to older participants, contrary to previous research [16, 44]. Nonetheless, during the COVID-19 pandemic, younger adults in the United States also reported more anxiety and depression symptoms than the general population, indicating increased need for mental health services [45]. In general, older adults have stronger coping skills and better resilience to stress, reflecting lessons learned with greater life experience [46]. Thus, younger adults may have had greater psychiatric need. Indeed, we found a significant indirect effect of SCS on the relationship between younger age and MH utilizer status. It should be noted, however, that our sample skewed particularly young, with 90% of the sample under age 30. Thus these findings might not generalize to a more age-diverse sample.

In terms of gender, self-identified cisgender women were also more inclined than self-identified cisgender men to utilize mental health services, which is consistent with prior research suggesting several mental health disorders are more common in women [47, 48]. Thus, the greater utilization of services might be attributable to greater need. Likewise, there was a significant indirect effect of SCS on the association between female gender and MH utilization status. However, attitudinal factors likely also play a role. Men tend to hold more negative attitudes towards the use of mental health services compared to women [44], which may lead to underutilization of such services [49, 50]. Addis & Mahalik [51] discuss that the common notions of “masculinity” require men to conceal their mental health needs to conform to their social role, likely making them more hesitant to seek professional help.

With regard to race and ethnicity, Asian participants were less inclined to utilize mental health services while Hispanic participants were more so, which contrasts with prior studies showing reduced help-seeking behaviors in both populations [52, 53]. Attitudinal barriers in the Asian and Hispanic populations, such as stigma towards mental health problems and treatment, have been found to be more prevalent and to hinder these populations from seeking professional help [54]. This trend has been observed in the National Latino and Asian American Study (NLAAS) which notes that even though Asian American individuals have a 17.3% lifetime risk of developing any psychiatric disorder, they are three times less likely to seek mental health services than White individuals [55]. Likewise, we did not see an indirect effect of lowered SCS on the association between Asian ethnicity and MH non-utilizer status, such that the reduced MH help-seeking intention in this subgroup was not attributable to lower emotional distress. The NLAAS also showed that only one-third of Hispanic individuals with clinical need use mental health services [56].

Nonetheless, the Latinx and Asian communities were under elevated health and financial stress during the pandemic because they are overrepresented in the essential workforce and thus unable to work remotely [57]. This may have affected them directly through their own occupational status as well as indirectly, through impact on friends and family. Asian individuals were under additional stress during this time because of the acute increase in anti-Asian crime and hate speech [58]. Likewise, Hispanic participants reported a higher prevalence of symptoms of anxiety, depressive, and COVID-19–related trauma- and stress-related disorders as well as suicidal behavior than non-Hispanic whites or Asian respondents [2]. Nonetheless, as with Asian American participants, we did not find an indirect effect of SCS on help-seeking intentions among Hispanic study participants.

Outside of cultural influences on barriers to care, there may also be protective cultural factors that influence both the incidence of mental health difficulties and associated help seeking behavior. For example, Asian Americans are almost 4 times less likely to die by suicide than are Native Americans and Alaska natives [40]. There is a large literature on the protective effect of social support with regard to psychological health in general and suicidality in particular [59–61]. Across Asian and Hispanic cultures, there is a greater influence of communal and familial affiliation than is typically found among White Americans [62]. According to 2021 U.S. Census data, Asian, Black and Hispanic Americans are more likely to live in multi-generational families than are White Americans [63]. In a survey of over 1500 adults living in multi-generational households, the majority described it as convenient or rewarding most of the time. Particularly for lower income households, the arrangements confer financial and caregiving benefits [63].

In our study, married participants were less likely to seek mental health services, which parallels the literature that shows that being partnered is a protective factor against the need for these services [61]. Relatedly, people who are married report higher levels of social support compared to those who are unmarried [64], suggesting their reduced usage of mental health services may be due to decreased need rather than barriers to care. Likewise, we found a significant indirect effect of SCS on the association between unmarried status and MH utilizer status.

In univariate analysis, participants with masters and doctorate degrees were also less likely to seek mental health services, consistent with other findings showing the negative correlation between educational level and need to use mental health services [65]. During the pandemic, individuals with high levels of education had the chance to work remotely while still guaranteeing financial security compared to individuals with lower education and socioeconomic status, who either had to attend their jobs in person, thus exposing themselves to COVID infection, or lost their jobs in the pandemic lockdowns [66]. However, this trend was not observed in multivariate analysis. This might suggest the interaction of educational status with other factors in our study or the overall high level of education in our sample. Likewise, our indirect effects analysis was not significant with regard to education.

In terms of work status, no significant difference was seen among all sub-categories. This does not parallel the literature which reveals that half or more of those who became unemployed during the pandemic developed mental health conditions such as anxiety and depression [67]. Our sample was mostly composed of White, educated and computer-literate participants who are likely to be financially comfortable. In addition, our sample contained a high number of students, likely less affected by the job market changes during the pandemic.

### Intention to utilize suicide prevention services

Suicide is one of the most severe consequences of mental health risk and hence warrants special consideration. Regarding participants’ intention to utilize SP referrals, many of the findings were consistent with those on mental health referrals. Similar results were noted regarding age, marital status, and education. We also found significant indirect effects of the SCS with regard to age and education and SP utilizer status, suggesting that increased suicidal risk (i.e., elevated SCS symptoms) contributed to these patterns of SP help-seeking intentions. Concerning gender, no significant differences were noted, which was unexpected. Prior studies have shown self-identified women to make the majority of calls to suicide hotlines worldwide [68]. Given the high level of lifetime SI within the sample, however, the self-selected nature of this sample may mask any effect of sex found in the general public.

In univariate analysis, our finding that Black participants were more inclined to seek suicide prevention services is not consistent with the literature [6]. This might be explained by the fact that African American communities have been disproportionately burdened with the COVID-19 pandemic, resulting in increased infection rates and number of deaths in the Black community [69]. In times of collective stress or adversity, such as a pandemic, suicidal behavior increases [70]; hence, utilization of suicide prevention services is likely to increase [71]. In both univariate and multivariate analyses, Hispanic individuals were also more likely to seek suicide prevention services, consistent with the endorsement of interest in pursuing mental health resources discussed above. The indirect effect analyses regarding SCS symptoms was not significant for either ethnic group, however, possibly related to low cell sizes, particularly for Black participants.

### Indirect role of SCS

As noted above, as a secondary analysis, we examined the role of the SCS as an explanatory variable between socio-demographic factors and mental health and suicide prevention resource utilization. There was a significant indirect effect of SCS on the relationship between age, gender, and marital status and stated intention to utilize mental health referrals and age, marital status, and education and intention to utilize SP referrals. This suggests that a number of the demographic patterns in stated help seeking intentions are due, at least in part, to increased psychological distress in these groups rather than simply being artifacts of attitudinal or structural barriers to care. That most of the models also yielded significant direct effects, however, suggests that other factors also play a role, such as the various demographically-relevant barriers/facilitators to care that have been extensively documented in the literature.

### Limitations and future directions

These findings should be considered in the context of several limitations. Our sample predominantly consisted of White, self-identified cisgender women, and English-speaking individuals residing in the United States. Future studies would benefit from a larger and more demographically diverse sample. As our study is based on self-report, response bias cannot be excluded. Future research incorporating other more alternative methods of measurement may produce stronger support for our results.

We utilized a convenience sample and so relied upon a self-selected sample of individuals particularly interested in completing a survey on mental health issues during the COVID pandemic. While this may limit generalizability, it might also be a strength as it likely highlights a population interested in mental health services. Additionally, this study only assessed responses in the first year of the pandemic and thus the identified patterns may not stay consistent across as the COVID-19 pandemic evolves over time. Nonetheless, the first year was the most acute and disruptive period in the United States.

Further, our study only assessed participants’ stated intention to utilize mental health and suicide prevention resources; thus we cannot know to what degree participants actually followed through with their stated intentions [72]. Finally, help-seeking behavior may have manifested in other ways besides seeking support or treatment from a mental health practitioner or any health professional.

However, within the context of these limitations, our study has several strengths. It utilized a large sample, addressed questions of critical import during a global crisis, and identified several sociodemographic groups with heightened need for mental health resources. Moreover, our study is to our knowledge the first to show an indirect effect of the SCS on the relationship between sociodemographic factors and help-seeking behavior.

## Abbreviations

MH: Mental health
SP: Suicide Prevention
SCS: Suicide Crisis Syndrome
SCI-2: Suicide Crisis inventory**-**2
